# Current Therapeutic Options for Alzheimer’s Disease

**DOI:** 10.2174/138920207783769549

**Published:** 2007-12

**Authors:** Alberto Lleó

**Affiliations:** Department of Neurology, Hospital Santa Creu i Sant Pau, Avda. San Antoni Mª Claret 167, Barcelona 08025, Spain

**Keywords:** Treatment, Alzheimer’s, acetylcholinesterase inhibitors, memantine, amyloid, behavioral symptoms, vitamin E, anti-inflammatory drugs.

## Abstract

Alzheimer’s disease (AD) is the most common neurodegenerative disease in the developed world. The increasing life expectancy in the last years has led to an increase in the prevalence of this age-related condition and has posed an important medical and social challenge for developed societies. The mainstays of current therapy for AD rely on the cholinergic hypothesis developed more than 20 years ago. These compounds, known as acetylcholinesterase inhibitors (AChEIs), inhibit the cholinesterases and aim at improving the brain synaptic availability of acetylcholine. These drugs have been approved for the treatment of AD based on pivotal clinical trials showing modest symptomatic benefit on cognitive, behavioral, and global measures. Memantine, an NMDA antagonist, has been recently included as a therapeutic option for AD. Memantine can be combined safely with AChEIs for an additional symptomatic benefit. During the last years our understanding of the mechanisms underlying the pathogenesis of AD has markedly expanded. Several putative neuroprotective drugs are thoroughly investigated and many of them have reached the clinical arena. It can be anticipated that some of these drugs will be able to slow/prevent the progression of this condition in the near future.

## INTRODUCTION 

Alzheimer’s disease (AD) is an age-related progressive neurodegenerative disorder, with onset usually in late life, characterized by cognitive impairment, a variety of behavioral symptoms, and restrictions in activities of daily living. AD is the most common form of dementia and the prevalence increases exponentially between 65 and 85, doubling every 5-year age group. Demographic predictions indicate that the prevalence is expected to increase if new preventive or neuroprotective therapies do not emerge [1, 2]. Risk factors for AD include age, family history of AD, female gender, lower education, cerebrovascular disease, vascular risk factors, prior head trauma, and presence of APOE-4 allele, among others. The cost of caring for patients with AD is extraordinary and this underlines the need for seeking new therapeutic approaches for this disease.

In this review we will focus on the current pharmacological options available in AD treatment: cholinesterase inhibitors, glutamate receptor modulation, vitamin E, antioxidants, anti-inflammatory agents and pharmacological management of behavioral symptoms. In the last section we will briefly summarize new therapeutic approaches for AD such anti-amyloid strategies [3, 4]. This manuscript will not cover other important aspects of the treatment, such as nonpharmacologic interventions, health maintenance activities, and caregiver support. 

## CHOLINESTERASE INHIBITORS

Acetylcholinesterase inhibitors (AChEIs) are the mainstays for treating AD and have become part of standard care according to the practice guidelines of the American Academy of Neurology [5]. Four AChEI drugs have been approved by the U.S. Food and Drug Administration (FDA) for the treatment of AD: tacrine, donepezil, rivastigmine, and galantamine. Tacrine is now rarely used because it has hepatotoxic effects in ~40 % of patients.

The use of the current available drugs in AD relies on the cholinergic hypothesis developed more than 20 years ago. The cholinergic hypothesis states that decreased cholinergic transmission plays a major role in the expression of cognitive, functional, and possibly behavioral symptoms in AD [6-8]. The cholinergic hypothesis is supported by several observations that show decreases in biochemical markers of cholinergic function in neocortex and hippocampus that correlate with dementia severity [9- 12]. Therefore, the hypothesis claims that cholinomimetic drugs can be beneficial to treat symptoms in AD. A limitation of the cholinergic hypothesis is the lack of cholinergic deficit observed in early stages of AD or in patients with mild cognitive impairment [13]. 

### Mechanism of Action of AChEIs 

**Fig. (1):**
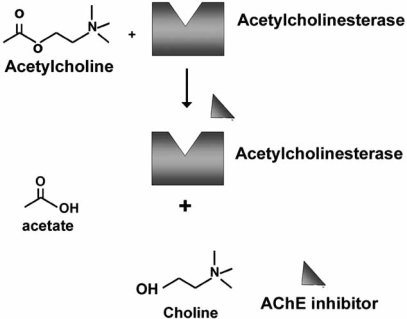
Hydrolysis of acetylcholine (ACh) by acetylcholine esterase.

The general mechanism of AChEI is to increase the brain availability of acetylcholine (ACh) through an inhibition of the enzyme acetylcholinesterase (AChE). ACh is produced in cholinergic neurons by the action of choline acetyltransferase, concentrated in vesicles, and released from the presynaptic cell following depolarization. Inhibiting AChE enhances cholinergic neurotransmission by prolonging the time ACh molecules remain in the synaptic cleft and are able to combine with muscarinic receptors. In mammals, two cholinesterases exist: AChE, which selectively hydrolyzes ACh, and butyrylcholinesterase, which hydrolyzes other choline esters in addition to ACh. The role of butyrylcholinesterase in humans is not completely understood. AChE has an active or catalytic center where the ACh is hydrolyzed releasing one acetic acid molecule and choline (Fig. **1**). All four approved AChE drugs inhibit this process by binding to the catalytic site although with minor differences. 

Cholinesterase inhibitors vary widely in their pharmacological profiles and affinities for AChE and butyrylcholinesterase. Donepezil and galantamine are 1000- and 50-fold, respectively, more selective for AChE than for butyrylcholinesterase, whereas rivastigmine inhibits both enzymes with similar affinity [14]. Galantamine also allosterically modulates nicotinic receptors. The clinical relevance of these differences remains unknown. 

### Clinical Pharmacokinetics of AChEIs 

The pharmacokinetic profile of AChEIs is summarized in Table **1**. Donepezil has a long half-life and does not require coadministration with food. It is metabolized by the cytochrome P450 enzymes CYP2D6 and CYP3A4 and may interact with drugs that inhibit these enzymes, such as cimetidine, ketoconazole, paroxetine, fluoxetine, and fluvoxamine. Galantamine exhibits 90%-100 % bioavailability and low protein binding potential [15]; coadministration of food may delay the rate of absorption but does not affect bioavailability. Since galantamine is also metabolized by cytochrome P450s, potent inhibitors of these enzymes may increase the cholinergic effects of the drug, leading to adverse effects [15]. Under these conditions patients with AD and moderate hepatic dysfunction may require a reduced dosage of galantamine. No dosage reduction is necessary in patients with renal insufficiency. Extended release capsules of galantamine are also available and should be administered once daily. Rivastigmine has a short half-life and is coadministered with food. It has a nonhepatic metabolism, and interactions are rare. A transdermal patch of rivastigmine has recently been launched and shows similar efficacy to capsules with fewer side effects. 

### Clinical Evidence Supporting the Use of AChEIs 

Tacrine was the first AChEI approved by the FDA for the treatment of AD but it is rarely prescribed now because the three newer drugs are equally effective and safer [16, 17]. Carefully conducted clinical trials of donepezil [18-23], rivastigimine [24, 25] and galantamine [26-28] all demonstrated small but statistically significant benefits on cognitive and global measures relevant to dementia. The magnitude of the effect in pivotal clinical trials was modest, on the order of 2.8-4.0 point improvements on the 70-point cognitive subscale of the Alzheimer’s Disease Assessment Scale (ADAS-Cog)[29], or 1-1.5 point improvements on the 30-point Mini-Mental Status Examination (MMSE) [30] compared to placebo over six months. Differences in global measures assessed by the 7-point Clinician Interview-Based Impression of Change scale [31] were on the order of 0.3-0.5 points in patients receiving the drug compared to patients receiving placebo. When evaluated according to these measures, the three commonly used inhibitors have similar efficacy. 

AChEIs appear to have a beneficial impact on the behavioral and neuropsychiatric symptoms as well, as suggested by a meta-analysis of well-designed clinical trials [32]. Most studies used the Neuropsychiatric Inventory [33] or the non-cognitive subscale of the ADAS to assess behavioral symptoms; in general, patients randomized to AChEIs had small but statistically significant improvements compared to placebo. Although in most studies data on specific symptoms are unavailable, improvement has been described in hallucinations, distractibility, aberrant motor behavior, and apathy. The use of AChEIs has also been reported to reduce caregiver burden and to delay nursing home placement [34-37]. 

Although AChEIs are the current standard of care for AD [5], there is less agreement on how long patients should be treated with AChEIs. The duration in most studies has been 6 months, and ethical considerations regarding the use of placebo limit the assessment of the long-term benefit of AChEIs in prospective studies [38]. Compared to the rate of decline predicted from historical untreated AD patients however, some studies have shown that the benefit may last at least four years [39-41]. There is no evidence on whether some patients respond better to one drug than another, or on the usefulness of switching from one AChEI to another. In general, switching is indicated based on side effects. Usually switching can be performed without a washout period, and interruptions longer than three weeks are not advised. There is no reason to combine two or more AChEIs, and this practice is not recommended. 

### Safety and Tolerability of AChEIs 

Overall, AChEIs are safe compounds, and side effects are generally limited to gastrointestinal symptoms (Table **2**). Less common side effects include sleep disturbances or acute confusion, which subsides with medication withdrawal. The incidence of these effects can usually be minimized by initiating treatment with a low dose and then escalating the dose slowly. The incidence of side effects is higher during initiation of treatment or during the dose-escalation phases. Another factor that affects the incidence of side effects is drug absorption rate. Drugs with short half-lives, such as rivastigmine and galantamine, are rapidly absorbed and may cause cholinergic side effects. Coadministration of either drug with meals delays absorption and can lower the incidence of these side effects. Donepezil has a longer half-life and does not require coadministration with food. 

### AChEIs in Clinical Practice

The three AChEIs in current use are relatively safe, easy to use, and well tolerated. The benefits they offer are also relatively modest, representing a small but measurable symptomatic improvement with no clear effect on rate of disease progression. Evident improvement in cognitive function is only observed in <10% of subjects. In counseling patients and caregivers on their use, it is reasonable to weigh these factors, along with the cost of the AChEIs. According to the American Academy of Neurology’s Practice Parameter evidence-based review [5], AChEIs are part of standard care in the United States, although a careful study by an English consortium concluded that the one AChEI studied (donepezil) was not cost-effective, as the benefits of treatment were below minimally relevant thresholds [42]. Patients/caregi-vers should understand the slight risk of nausea, vomiting or diarrhea (often limited to the first few days after initiating or increasing dose) and the likelihood that cognitive or behavioral improvements will be small compared to the overall course of the disease.

## GLUTAMATE RECEPTOR MODULATION: MEMANTINE

Another strategy to the treatment of AD is to block abnormal glutamatergic neurotransmission. Glutamate is the main excitatory neurotransmitter in the brain. One of its receptors, N-methyl-D-aspartate (NMDA), has been implicated in long-term potentiation, which is the neuronal mechanism responsible for learning and memory [43, 44]. Excessive glutamatergic neurotransmission leads to excitotoxity due to high intracellular concentrations of calcium, which causes neuronal dysfunction and death [45]. Memantine is a specific, low- to moderate-affinity, uncompetitive NMDA antagonist that was approved in 2004 by the FDA for the treatment of moderate to severe AD. Preclinical pharmacological studies suggested that memantine blocks the NMDA receptor and prevents calcium influx when neuronal firing rates are high, but leaves the calcium channel relatively open for neurotransmission at low stimulation rates [46]. Memantine was initially developed by researchers at Eli Lilly in 1963 and has been marketed in Germany and several other European countries since 1982 to treat a wide variety of neurological syndromes and cognitive dysfunction.

Despite the theoretical rationale for a neuroprotective mechanism for memantine, the studies supporting its use to date have demonstrated only symptomatic benefits. A 28-week double-blind placebo-controlled trial of memantine in outpatients with moderate to severe AD showed better functional and cognitive status in patients receiving memantine than in those receiving placebo [47]. The drug was well tolerated, and the patients on memantine did not report more adverse effects than those on placebo. In a separate small 12-week multicenter double-blind randomized trial, nursing home patients with dementia who took 10 mg of memantine showed better cognitive and functional scores than those on placebo [48]. Another study reported that the combination of memantine with donepezil seemed to be superior to donepezil alone in patients with moderate to severe AD [49]. This 24-week double-blind placebo-controlled trial showed that patients receiving the combination therapy had superior cognitive and functional scores, as assessed by the Severe Impairment Battery and the Activities of Daily Living Inventory, respectively. Recent trials have extended the benefit and safety of memantine to patients with mild AD [50]. In addition, the analyses of pivotal trials with memantine have indicated that the compound has beneficial effects on behavioral symptoms, as measured by NPI [51]. 

### Clinical Pharmacokinetics of Memantine 

Memantine is absorbed completely and has a half-life of 60-80 h. Memantine is not metabolized by the liver and therefore does not inhibit cytochrome P450. The pharmacokinetics of memantine is not altered by food, gender, or age. The recommended initial dose is 5 mg/day, followed by an increase of 5 mg/day every week until the maintenance dose (10 mg twice per day) is reached (see Table **1**). Like the AChEIs, memantine appears to offer AD patients modest symptomatic benefits with minimal adverse effects. In practice, given data on its potential incremental benefit in conjunction with donepezil, it is reasonable to consider adding memantine when a stable AChEI regimen has been achieved.

## VITAMIN E, ANTIOXIDANTS, AND ANTI-INFLAMMATORY AGENTS

Growing evidence for the existence of oxidative stress and the accumulation of free radicals in the brain of AD patients [52] has led to the notion of antioxidants as a potential treatment. The main antioxidant strategy in AD has been treatment with α-tocopherol (vitamin E). The principal clinical evidence comes from a double-blind randomized multicenter trial with α-tocopherol (2000 IU/day), selegiline (10 mg/day), both in combination, or placebo in patients with moderate AD [53]. The results of unadjusted comparisons showed no difference among the four groups after two years of treatment. However, when the baseline score on the MMSE was included as a covariate, a significant delay in the primary outcome was found with selegiline, α-tocopherol, and combination therapy. Treatment with α-tocopherol significantly delayed institutionalization. There was no improvement in cognitive tests in any of the treatment groups. The results of this study led many physicians to recommend high doses of vitamin E (2000 IU/day) to their patients. However, some concern has emerged after a recent meta-analysis suggested that high-dose (≥400 IU/day) vitamin E may increase all-cause mortality [54]. Excessive mortality was not detected in the large AD study [53], nor in a prior study comparing vitamin E to placebo in Parkinson’s disease [55]. Because of this new concern over mortality and because the putative benefits of high-dose vitamin E supplements are modest, many physicians have stopped prescribing vitamin E supplements or prescribe <400 IU/day.

Data from a three-year clinical trial that assessed the effects of vitamin E in patients with mild cognitive impairment failed to show a significant effect on slowing the progression to AD [56]. 

Ginkgo biloba (EGb 761) is a plant extract that is widely used by elderly individuals and patients with dementia in Europe and the United States to enhance cognition. In addition to putative antioxidant properties, the extract EGb 761 has been reported to reduce the aggregation of the amyloid-β peptide, which is widely implicated in the pathogenesis of AD [57]. The extract does not improve cognition in healthy elderly adults [58]. A 52-week multicenter placebo-controlled study showed a small improvement in patients with AD (average 1.7 points in the ADAS-Cog) [59]. Although physicians rarely prescribe ginkgo, it remains a popular remedy and is widely available in drugstores, supermarkets, and health food stores without a prescription. Although the efficacy of ginkgo biloba in treating dementia appears very small, most formulations appear safe. 

Retrospective observational studies have shown that use of nonsteroidal anti-inflammatory drugs (NSAIDs) may have a protective effect against the development of AD [60-65]. In a meta-analysis of nine studies, the use of NSAIDs was associated with a lower risk of developing AD, and the benefit was greater with long-term use than with intermediate use [60]. However, this possible protective effect does not extend to efficacy once AD symptoms have begun; treatment trials in patients with established AD reported no [66-69] or small [70, 71] benefit. A multicenter trial in AD with r-flurbiprofen conducted by Myriad is currently under way (www.clinicaltrials.gov). Currently, NSAIDs are not recommended as treatment for patients with AD. 

## MANAGEMENT OF BEHAVIORAL SYMPTOMS 

A majority of patients with AD develop behavioral disturbances during their illness course. These behaviors include aggression, agitation, hallucinations, delusions, sleep disturbances, depression, distractibility, apathy, aberrant motor behavior, and wandering. The presence of behavioral symptoms decreases the quality of life for patients and caregivers and increases the likelihood of institutionalization. When possible, the first step in treatment includes evaluation and treatment of factors (such as pain or fever) that may have triggered or contributed to the symptoms. Non-pharmacological interventions, such as music, light exercise, or relaxation should also be considered [72]. AChEIs and memantine have also been shown to moderate behavioral abnormalities, and this effect provides another basis for their use [32]. If the neuropsychiatric symptoms are severe or persistent, or cannot be controlled by modifying the environment, specific medications should be considered (Table **3**).

Anxiolytic drugs are often used to alleviate restlessness, pacing, agitation, and aberrant motor behaviors. In general, the doses should be as low as possible in order to avoid excessive sedation and motor impairment. 

Depressive symptoms and major depressive episodes commonly occur during the course of dementia and can affect up to 50% of patients with AD [73, 74]. Apathy is even more frequent, and it is often a diagnostic challenge to distinguish apathy from true depression. Many studies have tested the efficacy of antidepressants in treating the depression associated with AD yielding conflicting results. Some trials have shown some benefit with sertraline [73, 75], paroxetine, fluoxetine [76], citalopram [77, 78], imipramine [79], amytriptyline [76], clomipramine [80], and moclobemide [81] in AD patients with depression. Others [82-84] have found no differences between active drug and placebo. Possible explanations for the divergent opinions regarding treatment benefits include small sample sizes in some clinical trials, limited duration of treatment, different criteria and methods for measuring efficacy, and notable improvements in the placebo groups [85]. In practice, depressive symptoms in AD are usually treated with selective serotonin-reuptake inhibitors (SSRIs), beginning with low doses and increasing the dose based on clinical response and side effects (Table **3**). Tricyclic antidepressants have a similar efficacy but are used less often because most have prominent anticholinergic side effects, which have the potential to counteract the AChEI given as a treatment [76, 79]. 

Antipsychotic drugs are widely used to treat neuropsychiatric symptoms in AD, including delusions and hallucinations, when these symptoms become disturbing to the patient or the family. During the last decade, the newer atypical antipsychotic drugs (ie, risperidone, olanzapine, quetiapine, and aripiprazole, in order of introduction) have largely replaced the older conventional or first-generation antipsychotic drugs (eg, haloperidol and thioridazine) and have been considered preferred treatments for these behavioral disturbances associated with dementia. Although antipsychotic drugs are widely used, few randomized trials have evaluated their efficacy [86]. Risperidone, haloperidol, and olanzapine have been shown to be effective compared to placebo in AD [87-91]. One randomized placebo-controlled and two small open-label nonrandomized trials have shown that quetiapine may provide some benefit in AD patients [92-94]. Quetiapine did not worsen motor symptoms in patients with dementia and parkinsonism [95]. A recent double-blind, placebo-controlled trial suggested that the efficacy of atypical antipsychotic drugs in AD is limited by the presence of adverse effects [96]. There are no sufficient data on other newer drugs, such as sertindole, aripiprazole, zotepine, or ziprasidone. Two trials directly compared risperidone versus haloperidol; although there were no differences in global neuropsychiatric scores, risperidone was significantly better at suppressing aggressiveness [90, 97]. When medication is required, atypical antipsychotics are preferred because of the lower incidence of extrapyramidal side effects such as parkinsonism and tardive dyskinesia, compared with conventional antipsychotics such as haloperidol (Table **3**). A concern is that the use of atypical antipsychotics has been associated with an increased risk of adverse cerebrovascular events and death. A recent meta-analysis of 15 placebo-controlled studies (9 unpublished) suggested that the use of antipsychotics was associated with small increased risk for death (OR = 1.54) compared with placebo [98, 99]. It is important to mention that no individual drug is responsible for the effect, and it is only when all trials are combined that a significant effect is found. In practice, the use of antipsychotics should be prescribed with care in patients with dementia, in low doses and rapidly discontinued when symptoms resolve. 

## FUTURE PERSPECTIVES

Molecular mechanisms of AD are increasingly well understood. There is growing consensus that generation of Aβ oligomers is a key event in the pathogenesis of AD and initiate a cascade of events eventually leading to synaptic and neuronal dysfunction. Just as the cholinergic hypothesis of memory loss guided drug development that eventually led to the introduction of AChEIs, drug discovery programs guided by the amyloid hypothesis hold the promise of discovering compounds that will alter the underlying pathophysiology of the AD process. 

The two main approaches to anti-amyloid therapy have been to reduce the production or to increase the clearance of Aβ_42_ [100]. The goal of Aβ reduction has centered on a search for molecules that inhibit β- and γ-secretase. The search for γ-secretase inhibitors has yielded several potent compounds that inhibit γ-secretase in cell lines and dramatically lower Aβ levels in blood, cerebrospinal fluid, and brain [101]. Enthusiasm for developing γ-secretase inhibitors has now cooled somewhat, since γ-secretase also process other substrates, including the Notch receptor [102] involved in critical pathways. Indeed, *in vivo* treatment with a γ-secretase inhibitor reduced Aβ production but also caused profound alterations in thymocyte differentiation and other Notch-dependent processes [103]. A few small clinical trials using γ-secretase inhibitors have been conducted, and the compounds decreased Aβ in plasma but not in the cerebrospinal fluid [104]. β-secretase [105, 106], is another potential target for drug development [107, 108]. Clinical data support this line of drug development, as β-secretase activity in human brain increases with age [109]. Developing a β-secretase inhibitor, however, has proved challenging, and none has been tested extensively in humans. 

The main approach to induce Aβ clearance has been anti-amyloid immunotherapy. Since the surprising discovery that immunization with Aβ_42_ prevented the appearance of amyloid pathology in a transgenic mouse model of AD [110], other studies have reproduced the results using different models [111, 112]. Passive immunization with antibodies against human Aβ also decreased Aβ in transgenic mice and improved performance in test behaviors [113, 114]. Based on these preclinical findings, a multicenter randomized double-blind placebo-controlled Phase II trial was organized to test the safety and efficacy of active Aβ_42_ immunization in humans. The immunization trial was halted after the second injection because 6% of patients who received the active immunization developed meningoencephalitis [115]. Although this initial trial did not proceed as smoothly as anticipated, the trial yielded important findings that validate the immunological approach to treat AD. Neuropathological evaluation of immunized cases showed areas with unusually reduced amyloid burden and evidence of Aβ-associated microglia, suggesting that the immunization had increased Aβ clearance by activated microglia [116, 117]. Based on these observations, Elan and other companies have launched new trials that are currently ongoing with passive or safer active immunization in patients with AD. 

Another approach to stimulate Aβ clearance is to develop compounds that bind to Aβ. One example, is tramiprosate, an antifibrillization agent tested in AD. Although in the phase II study the drug was shown to be safe and was able to lower Aβ_42 _levels in CSF, the phase III study was halted due to lack of efficacy [118]. 

## CONCLUSIONS

AChEIs and memantine are the main available agents prescribed for treating the cognitive symptoms in AD. These drugs produce modest symptomatic benefit on cognitive, behavioral and functional symptoms with minimal impact on the disease process. AChEIs can be combined safely with memantine for an additional symptomatic benefit. Several putative neuroprotective drugs are thoroughly investigated, and the development of interventions that substantially delay the onset or modify the progression of Alzheimer’s disease can be anticipated.

## Figures and Tables

**Table 1.** Summary of the Pharmacokinetic Profiles of the AChEIs and NMDA Receptor Antagonist Used in AD

Drug	Bioavailability (%)	T_max_(h)	Elimination Half-Life (h)	Hepatic Metabolism	Drug Interactions
Donepezil (Aricept^®^)	100	3-5	60-90	CYP1A2, CYP2D6	Cimetidine Ketoconazole Paroxetine Fluoxetine Fluvoxamine
Rivastigmine (Exelon^®^)	40	0.8-1.7	2	nonhepatic	rare
Galantamine (Reminyl^®^)	90-100	0.5-2	5-7	CYP2D6, CYP3A4	Paroxetine Fluoxetine, Fluvoxamine Ketoconazole Erytromycin
Memantine (Namenda^®^)	100	3-7	60-80	nonhepatic	rare

**Table 2.** Management of the AChEIs and NMDA Receptor Antagonist Used to Treat Cognitive Loss in Patients with AD

Drug	Initial Dose	Maintenance Dose	Common Side Effects	Uncommon Side Effects
Donepezil (Aricept^®^)	5 mg daily for 4-6 weeks	10 mg daily	nausea, diarrhea, vomiting	insomnia, bad dreams, dizziness
Rivastigmine (Exelon^®^)	1.5 mg b.i.d. for 2 weeks. Increase 1.5 mg per dose every 2--4 weeks	3-6 mg b.i.d.	nausea, diarrhea, weight loss, vomiting	dizziness, fatigue, headache
Galantamine (Reminyl®) (Reminyl ER)	4 mg b.i.d. for 4 weeks. Increase 4 mg per dose every 4 weeks (or 8 mg of the extended release formulation)	8-12 mg b.i.d.(or 16-24 mg of the extended release formulation)	nausea, vomiting, diarrhea, dizziness	Weight loss, headache, abdominal pain, asthenia, somnolence
Memantine (Namenda^®^)	5 mg/day. Increase 5 mg every 2 weeks.	10 mg b.i.d.	hallucinations, confusion,dizziness, headache	Tiredness

**Table 3.** Summary of the Main Agents Used to Treat Neuropsychiatric Symptoms of AD

Type and Drug	Initial Dose	Maintenance Dose	Targeted Symptoms
*Atypical antipsychotics* Risperidone Olanzapine Quetiapine Ziprasidone	0.5 mg 2.5 mg 25 mg 20 mg	0.75-2 mg/day 5-10 mg/day 100-300 mg/day 40-160 mg/day	psychosis and agitation
*Traditional neuroleptic * Haloperidol	0.25 mg	1-3 mg/day	psychosis and agitation
*Selective serotonin-reuptake inhibitors* Fluoxetine Sertraline Paroxetine Citalopram Escitalopram	5 mg 25 mg 10 mg 10 mg 5 mg	10-40 mg/day 75-100 mg/day 10-40 mg/day 10-20 mg/day 20-40 mg/day	depression, anxiety, agitation
*Anxiolytics* Diazepam Alprazolam Lorazepam	2.5 mg 0.5 mg 0.5 mg	2.5-10 mg/day 0.5-2 mg/day 0.5-2 mg/day	anxiety, restlessness, insomnia
